# Exploring patients’ lived experience on the barriers to accessing low back pain health services

**DOI:** 10.4102/phcfm.v14i1.3523

**Published:** 2022-12-19

**Authors:** Morris Kahere, Khumbulani Hlongwana, Themba Ginindza

**Affiliations:** 1 Discipline of Public Health Medicine, Faculty of Health Sciences, University of KwaZulu-Natal, Durban, South Africa; 2 Cancer and Infectious Diseases Epidemiology Research Unit (CIDERU), School of Nursing and Public Health, University of KwaZulu-Natal, Durban, South Africa

**Keywords:** chronic low back pain, barriers, access, diagnostic, CLBP treatment

## Abstract

**Background:**

The burden of chronic low back pain (CLBP) is a major concern to public health. However, the treatment of CLBP in primary care has shown to be ineffective in South Africa. Understanding the barriers encountered by patients in accessing CLBP healthcare services is paramount in the development of context-specific intervention strategies.

**Aim:**

To explore the patients’ lived experiences on the barriers to accessing diagnostic, referral and treatment services for CLBP.

**Setting:**

A health facility-based study conducted at five primary public hospitals in KwaZulu-Natal, South Africa.

**Methods:**

A phenomenological study by means of in-depth interviews using the general interview guide approach. Interviews were conducted by a research assistant with relevant experience and qualifications in qualitative methods. A total of 15 participants were recruited to participate in this study. All interviews were audio-recorded and transcribed. Data were analysed iteratively until saturation was reached, where no new themes were emerging. All the transcripts were exported to NVivo 12 Pro for analysis.

**Results:**

The results of this study identified the following barriers: travel, long waiting periods, shortage of personnel, poor infrastructural development, inadequate healthcare personnel, communication barrier, social influence, beliefs around cause and effect, misdiagnosis and inappropriate and/or ineffective treatment approaches.

**Conclusion:**

This study concluded that barriers to patients’ accessing diagnostic, referral and treatment services exist. Efforts should be made towards developing health systems in underserved communities.

**Contribution:**

This is the first study to be conducted in South Africa that explored the barriers associated with accessing healthcare services for chronic low back pain. Based on the results of this study, in order to improve health outcomes for low back pain there need to be a change of emphasis in primary health care by ensuring sufficient allocation of resources towards musculoskeletal disorders.

## Background

Chronic low back pain (CLBP) is increasingly becoming a major global public health concern, constituting a significant economic burden.^1^ Globally, the experience of low back pain (LBP) is common across all age groups.^1,2^ Low back pain is a heterogeneous condition with multifactorial aetiologies,^1,2^ which is why the traditional biomedical treatment approach has shown limited effectiveness.^3^ Guidelines recommend the use of a biopsychosocial model in primary care, an approach that is yet to be fully recognised in low and middle-income countries (LMICs).^4^ While many LBP cases resolve within a few weeks with no or minimal intervention, most presentation will have episodes of recurrent pain or disability and require a targeted, multidisciplinary treatment approach.^5^ Thus, LBP is now viewed as a chronic, recurrent condition, which is a departure from the previous ideas of categorising it to acute, subacute or chronic LBP.^5,6^

Low- and middle-income countries including South Africa are faced with many healthcare challenges, with the primary focus directed towards communicable diseases and child and maternal health, which makes it difficult to reduce the burden of non-specific CLBP.^7^ Evidence has shown that chiropractic is by far the best in the management of non-specific CLBP.^8^ However, in South Africa chiropractic is only available through private means and its incorporation into the national public health system is still a subject of debate despite numerous evidence of its effectiveness in then management of musculoskeletal disorders. A recent scoping review of studies conducted in sub-Saharan Africa indicated that there is a paucity of CLBP prevalence data in this context.^9^ Funding for CLBP research is inadequate and additionally the proportion of chiropractors to patients in South Africa is significantly large. Training of chiropractors in South Africa is insufficient and they are difficult to access.^10^ This leads to delays in appropriate treatment and further contributed to disability, as the window of opportunity to start treatment will have been missed.^7^ With South Africa falling in the LMIC category, the majority of citizens are low-income earners who cannot afford private healthcare (chiropractic).

Considering the burden associated with CLBP, including poor psychosocial wellbeing, disability, production loss, suboptimal performance and increased societal and health economic costs, effective and reliable healthcare services for CLBP should be readily accessible.^11^ In order to implement effective CLBP patient-centred healthcare services, it is important to understand the patients lived experiences on the barrier to accessing healthcare services, including diagnostic, referral and treatment of CLBP. The identification and elimination of these barriers is pivotal to CLBP patients, healthcare providers, policymakers, funders, stakeholders and other involved actors in the planning of healthcare programmes in order to improve service delivery, and CLBP health outcomes and thereby reducing the burden thereof.^12^ Through exploring the patients’ views on CLBP, this study aimed to generate evidence regarding barriers encountered in the primary healthcare system.

## Methods

### Study design

This was a qualitative study based on a descriptive phenomenological design to determine the burden of CLBP among adults in KwaZulu-Natal, South Africa. This was part of a large multi-methods study conducted to determine the burden of non-specific CLBP among adults in KwaZulu-Natal, with estimates on prevalence and risk factors,^13,14^ factors influencing CLBP disability,^15^ economic burden^16^ and barriers to care. This study is reported in accordance with the consolidated criteria for reporting qualitative research.^17^

### Research team and reflexivity

The principal investigator (M.K.) was a male Doctor of Chiropractic (DC) and a PhD student, supervised by a qualitative research expert (K.H.). Interviews were conducted by a research assistant (A.G.) who was also a PhD student, and a holder of a master’s degree in Media Studies and a bachelor’s degree in Public Relations. A.G. had training and experience in conducting in-depth interviews for qualitative research. Both the principal investigator and the research assistant had no prior relationship with the participants.

### Study setting

Participants were recruited from five provincial hospitals (viz. Prince Mshiyeni Memorial Hospital, Mahatma Gandhi Memorial Hospital, Addington Hospital, Hillcrest Hospital and Clairwood Hospital) in KwaZulu-Natal. These hospitals are located in the eThekwini District Municipality, which is the most populous district in KwaZulu-Natal province. KwaZulu-Natal is a South African coastal province with a population of 11.4 million people, constituting about 19.6% of the total South African population, with a geographical area of approximately 94 361 km^2^.^18^

### Participants’ selection

An initial random sampling of the study sites was conducted using the hat method. A purposive heterogeneous and criterion-based sampling techniques were used to recruit participants that were anticipated to be knowledge-rich. The nurses in charge were instrumental to assisting with the identification of potential participants. The interviewer approached potential participants face-to-face in a hospital setting, explained the purpose of the study and invited to participate those who showed interest. The following criterion was used to identify knowledge-rich participants: Adult participants (≥ 18 years) who had experienced CLBP which persisted for 12 consecutive weeks and above in the past 12 months and have been through the primary health care (PHC) clinics or community health centres (CHC) for CLBP care services and understand the nitty-gritty of the processes and procedures in those PHC/CHC centres. Patients that have been referred to a district hospital and experienced both inpatient and outpatient care for CLBP at a regional or tertiary hospital and have faced some challenges or frustrations with an in-depth understanding of the referral system that is based upon their lived experience were also considered knowledge-rich and were invited to participate. The heterogeneity of the sampling was based on the patients’ demographic and socioeconomic profiles and these patients were recruited from five different health facilities. A human body drawing was used to describe the anatomical boundaries of LBP and CLBP was defined as pain or muscle stiffness or tightness below the costal cartilages and above the inferior gluteal fold with or without leg pain, lasting for 12 weeks and above.^19^

### Sample adequacy estimation

In qualitative research sample adequacy is determined by saturation, which is a point in data collection when no additional issues or insights emerge from data and all relevant conceptual categories have been identified, explored and exhausted.^20^ A study by Hennink et al.^20^ who investigated code saturation versus meaning saturation and identified how many interviews are required to achieve such, showed that code saturation was reached at nine interviews. In this study, it was anticipated that data saturation will be reached at 10 interviews. In this study data was collected in an iterative manner until saturation was reached. Saturation was, therefore, reached at 15 participants.

### Study instruments

In-depth interviews using the general interview guide approach were used to collect data exploring patients’ perspectives on the barriers to LBP access, diagnosis, referral and treatment. The interview questions were adapted from a similar published study done on healthcare worker perspective; however, this was tailored to patients’ perspective. This guide was pilot tested among three CLBP patients, the principal investigator, the interviewer and an expert in qualitative research methods. These three patients did not form part of the main study participants. Relevant changes were made prior to the commencement of the final data collection process.

### Data collection

The data generation process followed the published guidelines established in conducting in-depth interviews and is described in detail elsewhere.^21^ Data were collected in December 2021 during the coronavirus disease 2019 (COVID-19) pandemic. The interviewer made use of the personal protective equipment which was supplied by the participating hospitals in line with the national guidelines to mitigate the spread of the SARS-CoV. Following the identification of knowledge-rich participants, the research assistant (A.G.) approached them face-to-face, introduced herself and explained the purpose of her visit. Those who showed interest were invited to participate and were taken to a quiet room (where there was no disruption and/or interruption) where the interviews took place and explained the process in detail including the anticipated duration of the interview (which was approximately 45 min). The associated risks and potential benefits were highlighted in detail before consent. Nevertheless, the study involved no human harm as all the participants’ identifying information was protected through a coding process where each participant was given a unique identification number that was only used for the purpose of the study. After all the information that pertains to the research was clearly explained to the potential participants, everyone was given an opportunity to ask questions. Following that and after the participants have understood the research process and the terms and conditions, those that were willing to participate were required to consent in writing prior to the commencement of the interview process and recordings. The interviews were audio-recorded while at the same time the interviewer (A.G.) was making some field notes of important points in order to identify areas of further probing and follow-up. Non-verbal language such as body language was also noted and recorded during the interview process. Data were collected iteratively, where one interview was conducted per day. At the end of the interview process, all audio-recorded data were transcribed by a professional transcriber. Those interviews conducted in isiZulu were initially transcribed and then translated into English. Only about 5% of the interviews were back-translated by another independent professional translator to ensure consistency and that no meaning was lost in translation. All transcripts were returned to the participants (interviewees) for comments or correction before the final analysis. At the end of each interview day, debriefing meetings were held between the researcher and the interviewer, where the proceedings of the day were discussed. All audio-recorded data, transcripts and notes were thoroughly analysed to identify emerging themes and areas where further probing was required. This process was conducted in an iterative manner where the research team were concurrently sampling, collecting data and analysing data.^22^ This iterative process enables ‘theoretical sampling’, which involves identifying concepts from data that are used to guide participant recruitment to further explore those concepts in subsequent data collection until theoretical saturation was reached.^22^

### Data analysis

All the interview recordings were transcribed verbatim by the research assistant, checked by the principal investigator and then exported to NVivo 12 Pro (QRS International, Burlington, Massachusetts, United States [US]) data management software for analysis.^23^ A three-step systematic process was used to analyse the data:^24^

- Firstly, the NVivo 12 Pro was used to scan through the primary data to identify terms or phrases that were most commonly used or repeated by respondents, relevant to the purpose of the study. These terms or phrases were highlighted and organised into codes, which were then used to code all the transcripts.
- Secondly, the coded data were reviewed to identify patterns and relationships where related/similar codes were combined or organised into categories.
- Finally, the coded data were then interpreted to generate themes. Thematic content analysis was used to give a narrative account of the themes that emerged.

The data were then summarised, linking the findings to the purpose of the study. Findings of this study and the methodology used were then compared with the findings of the literature review and the differences were discussed. The coding process was conducted by two of the authors (M.K. and K.H.) who were working separately, using two different coding methods, NVivo and manual based coding. The results were compared and differences discussed by all the three authors until agreement was reached.

### Ethical considerations

University of KwaZulu-Natal Biomedical Research Ethics Committee (BREC) (ref no. BREC/00000205/2019) and the KwaZulu-Natal Department of Health Ethics (ref no. KZ_201909_002).

All participants were told that they can withdraw from the study at any juncture if they wish not to continue. All participants who volunteered to participate and fulfilled the criteria for inclusion were required to consent in writing after they have understood the terms and conditions of the study prior to the actual data collection process.

## Results

### Demographic characteristics

The study constituted 15 adult participants (Table 1) with a two to three male to female distribution ratio. The average age of the participants was 50 years. More than half of the participants were experiencing CLBP, two-fifth of them were severely disabled and a little below a third were crippled because of LBP.

**TABLE 1:** Characteristics of study participants (*N* = 15).

Participant ID	Sex	Age	Ethnicity	Education	Occupation	LBP duration
P001	Female	43	Black African person	Secondary	Cleaner	CLBP
P002	Female	56	Indian person	Primary	Housewife	CLBP
P003	Male	49	Black African person	Primary	Factory worker	CLBP
P004	Male	42	Black African person	Secondary	Factory worker	CLBP
P005	Male	46	Black African person	Secondary	Factory worker	CLBP
P006	Male	54	Indian person	Secondary	Automotive sales	CLBP
P007	Female	55	Black African person	Primary	Security guard	CLBP
P008	Female	63	Black African person	Primary	Housewife	CLBP
P009	Female	43	Indian person	Tertiary	Registered nurse	ALBP
P010	Male	37	Black African person	Secondary	Security guard	SALBP
P011	Female	39	Black African person	Tertiary	Bank teller	CLBP
P012	Female	57	Black African person	Secondary	Street vendor	CLBP
P013	Female	61	Black African person	Primary	Retired	CLBP
P014	Female	54	Indian person	Primary	Street vendor	CLBP
P015	Female	49	Black African person	Secondary	Cleaner	CLBP

CLBP, chronic low back pain; LBP, low back pain; ALBP, acute low back pain; SALBP, sub-acute low back pain.

### Overview of the emerged themes

The results of this study were deductively packaged into four key thematic content areas, namely: (1) access, (2) diagnostic, (3) referral, and (4) treatment services in primary care as shown in Table 2. These four main themes were pre-identified and yielded 13 subthemes presented in Table 2 as a summary of the key study findings.

**TABLE 2:** Key findings and potential solutions – Patients’ perceived barrier.

Main themes	Sub-themes
Accessing facilities	Travel burden to the health facility
	Long Long queues of waiting
	Shortage of skilled personnel
	Pyramidal pathway-induced delays and unavailability of certain categories of professions
	Unequal access to quality healthcare services
	Sociocultural beliefs around cause and effect
Referral	Inefficient or ineffective process
	Insufficient knowledge
	Social influence
Diagnostic services	Inadequate equipment
	Shortage of skilled personnel
Treatment services	Inappropriate treatment
	Poor communication

#### Theme 1: Access

Access to healthcare services is defined by the Institute of Medicine as, ‘having timely use of primary healthcare services to achieve optimum health outcomes’.^25^ Six subthemes emerged as important barriers to accessing PHC services.

**Subtheme 1.1: Travel burden to the health facility:** The burden of travel to and from the healthcare facility emerged as one of the most concerning barriers to accessing healthcare facilities, especially because outpatients were often booked for multiple visits to the facility for follow-up treatments or because pain was not going away. The lack of efficient emergency transportation services (ambulances) exacerbated the situation, thereby forcing already financially struggling patients to hire private vehicles:

‘I borrowed money to hire a car and was put on a stretcher, that time it was hard to even sleep or stand, I just couldn’t do anything. There is a hospital nearby, usually takes about 45 or so minutes to walk but I couldn’t walk as I was unable to.’ (P001)

**Subtheme 1.2: Long queues of waiting:** Long waiting periods arising from high patient volume and the fact that these facilities are not specialised meant that everyone (long queues of women coming for their family planning pills, human immunodeficiency virus (HIV) patients and children’s reviews) wait to be served by the same personnel. Everyone goes through the same process and must wait for their turn, as expressed by one participant:

‘Yah! I went to the clinic, but the queue was long, and I was in so much pain that the length of the queue was even getting me angry. I almost felt like crying in the middle of the queue and the nurses were working very slow, like there were on a strike or something because there were taking a lot of time with one person. At first, I thought maybe there were busy doing investigations of somethings, but after several hours of waiting, my turn came and realise that there were just talking to each other and not considering the people waiting to be served.’ (P002)

**Subtheme 1.3: Shortage of skilled personnel:** Primary health care clinics and CHC in underserved communities such as the rural areas suffer a significant shortage of skilled personnel, thereby, resulting in limited access to quality multidisciplinary healthcare. Another important issue raised by one of the respondents is that most community health workers are poorly trained, some of which from institutions that are not accredited by the higher education. One respondent, said,

‘Honestly, some of our community health workers are not qualified enough to deliver the best care possible. This is caused by the fact that, most of our brilliant children when they pass school, they want to go to better places where they can grow professionally, while leaving us with no one willing to help us, therefore, those who did not do well in their high school or those who have supplemented, are the ones that goes to these unaccredited institutions to get trained as community health workers. That is why we do not have access to quality care … And I think this can be solved by developing the infrastructure of our local clinics so that they can attract better qualified workers. Also, if the government or non-goverenmental organisations (NGO’s) can provide sponsorship for people to work in these communities and provide them with competitive salaries and living conditions.’ (P003)

**Subtheme 1.4: Pyramidal pathway-induced delays and unavailability of certain categories of professions:** Unavailability of certain categories of healthcare professionals is an important barrier to the access and uptake of healthcare services, as shown in this study. The most reported service that is not easily accessible in PHC was physiotherapy and rehabilitation services. One respondent said:

‘Sometimes I went to the healthcare facility, and they told me that we are waiting for a physiotherapist to be deployed at the facility and it’s been one year now, and no one has arrived yet. So, they had to refer me to another community health centre in a different community where I went and still found no one and mind you, I was in need to help and now I am just being tossed around”. Eventually, I had to bypass the referral system and went straight to the district hospital and told them all that I had gone through and unfortunately the sister there referred me back to the CHC to get a referral letter as she was saying they only accept referrals and not just walk-ins unless if it’s an emergency and is brought by an ambulance.’ (P004)

**Subtheme 1.5: Unequal access to quality healthcare services:** Differences in socioeconomic status render unequal access to quality healthcare services in South Africa where insurance coverage is not universal. Only those with medical aids have options to access any other health facility even private healthcare. Those individuals with no means of medical cover will have to go to the public health sector which is not equipped enough (in terms of medical professionals and infrastructure) to deal with the masses of people that cannot afford medical aid. Thus, there is a divide in the provision of healthcare between the public and private sector, where the private sector is not easily accessible to the uninsured. One respondent said:

‘I was given a referral letter and I knew that what I want is not available at my district hospital but at the local private hospital. I wanted to get an magnetic reasonance imaging (MRI) scan and it was only available at one of the private hospitals in the entire district, however, due to lack of money, I ended up just holding onto my letter and not getting any help. However, if only I had insurance, I would have the option to go to the private hospital and get helped there. So, I would say lack of medical aid is a big problem because it limits your options of accessing good quality health care.’ (P005)

Thus, the cost variable should be dealt with by the responsible decision and policy makers to ensure health equity and make sure that there is equal access to quality affordable healthcare. One of the participants mentioned that:

‘For you to have access to quality health care, you need to be rich or have a good job where they can pay for your medical insurance for you to be treated anywhere, even in private practice. For example, my job doesn’t pay for my medical aid and what I get paid is not enough for me to pay for the medical aid, because I have 5 children and they are all going to school and different levels and I and supposed to feed them and make sure there are dressed, pay their school fees, buy them books and all that. So, by the time your salary hit your bank account, it is already allocated, and nothing is left.’ (P006)

**Subtheme 1.6: Sociocultural beliefs around cause and effect:** In KwaZulu-Natal (KZN), LBP is regarded as a trivial condition that resolve on its own and thus people are neglect to seek care with hope that the symptoms will eventually fade away without any intervention or by the use of over-the-counter medication. This attitude negatively affects the prognosis of acute episodes of LBP because of a poor early treatment-seeking behaviour, especially if the pain is not severely disabling, as articulated below:

‘I first asked because before I had LBP, I had been vaccinated so I asked the sister at the hospital where I work, and she said its back pain that will go away on its own as times goes on, but it nothing changed. She advised me to buy pills, I think they were called Mobic tablets that would stop the pain, but they didn’t work, that was November 24.’ (P007)

The patient’s behaviour in following through the referral process is another observed barrier.

#### Theme 2: Referral

According to the definition of the World Health Organization (WHO), ‘referral can be defined as a process in which a health worker at one level of the health system, having insufficient resources (drugs, equipment, skills) to manage a clinical condition, seeks the assistance of a better or differently resourced facility at the same or higher level to assist in, or take over the treatment of the patient’s case.’^26^

The result of this study shows that CLBP patients encounter challenges as they follow through the hierarchy of the referral system. Three subthemes emerged as major barriers to effective and efficient referrals.

**Subtheme 2.1: Poor technological advancement:** Several delays have been noted in the referral system in KZN because of the use of the traditional paper-based referral system, which also makes it difficult to follow-up on patients. Paper-based referral system poses many challenges that include chances of losing the referral letter, ineffective internal or inter-institutional communication with regard to referrals, thus no accountability as the system solely relies on physically hand-held referral letters. This further exacerbates the duration of waiting to receive care. One of the respondents said:

‘So, when I was referred I was given a letter from the PHC to come here at the district hospital, so I went home because I had waited for a long time at the clinic and the time had gone, so I wanted to go to the hospital the following day in the early morning so that I can be one of the first people to be served as I don’t like waiting too much. When I got up the following morning, I just couldn’t find that letter, and was not sure if I had misplaced it in the house or lost it the previous day when I was coming home. That got me worried because if it was lost, it means anyone can have access to my confidential information in that letter, including my personal details. However, I had to go back and wait in another queue to get another referral letter for me to proceed to the hospital. When I arrived at the hospital, I though those with referral letters will be served first or they will be prioritised but unfortunately, that wasn’t the case. I had to wait there for almost five hours before I was served by the nurse. It took me another two hours to be able to see the doctor and most of the time was lost in waiting.’ (P008)

The referral system is termed a system because it encompasses several aspects that include health system issues, initiating facility, referral practicalities, receiving facility, supervision, capacity building and continuous quality improvements. Therefore, an effective referral system minimises unnecessary patient follow-ups, increases quality, reduces waiting time, increases access, efficiency, the number of referrals, and confidentiality of patient information, improves the relationship between first level and specialised care and increases safety for patients and consequently, enhances efficacy.

**Subtheme 2.2: Insufficient knowledge about the referral system:** This study found that the majority of CLBP patients lack sufficient knowledge about the referral system. Most patients seek care at their nearest healthcare facility with the hope of getting sufficient services they require to manage their CLBP symptoms. However, because of the deficiency of information about the referrals, often patients are dissatisfied when they feel like they are being tossed from one facility to another as was highlighted by one of the respondents:

‘When I went to the clinic, it was actually my first time experiencing back pain and as I always see people at the clinic getting some medication, I thought I should quickly go to the clinic to get help because I was in excruciating pain that was shooting down to my right leg and I could not stand up straight. My cousin tried to call an ambulance, but the ambulance never showed up and he ended up hiring a private vehicle. When we got to the clinic, I was with hope that I will be attended to quickly and will be able to feel better. However, that was not the case, we had to wait in the queue and waiting for about an hour or two, the nurse told us that we need to get an x-ray before they can do anything. So, she gave me a letter to take to the hospital to get x-rayed, and I was disappointed and frustrated because they could have told us before we wasted our time in the queue. When we went to the nearest hospital, they told us that they only accept patients from the district hospital. This district hospital was far away, but with no option we had to drive there. When we got to the district hospital, the nurses said you need to first book an appointment then do the x-ray and you cannot book an appointment and do the x-ray on that very same day, you will need to come the next day or any other day … Has we known this before, we could have just called the district hospital and booked the appointment without wasting time and money.’ (P009)

**Subtheme 2.3: Social influence:** There are certain instances where our social circles dictate or influence what should be done. A lack of social and/or familial support is another big hindrance to accessing referral systems. One participant said:

‘I was referred to the hospitals, but my cousin discouraged me to go there because he was saying you are weak, this is just low back pain, and you need to eat a lot of cow legs [*amancina, in isiZulu*] and other traditional foods to make your back strong. So, I listened to that and did not go to the hospital. My back kept on getting worse and worse until I realise that I don’t need to listen to anyone because it’s me who is experiencing this pain, I know how severe it is and if I keep listening to people who are not in pain, they will let me die so no no no. So, what I am basically saying is, bad friends can also be a problem that can disrupt a good system with their bad influence.’ (P010)

#### Theme 3: Diagnostic services

Accurate diagnosis is key to effective treatment in primary care. Because of the non-specific nature of LBP, it is often mismanaged, and current guidelines have already shown that the treatment of LBP in primary care is ineffective. Two subthemes emerged as the main barriers in accessing quality diagnostic services.

**Subtheme 3.1: Inadequate equipment:** The use of inadequate diagnostic equipment poses a significant hindrance to accurate diagnosis and administration of appropriate treatment plan. Limited resources in this setting, where a large population relies on a single X-ray machine. One woman said:

‘Here in Umlazi we have got only one hospital, yet we are a very large community. If only we have at least three hospitals like the one, we have got maybe it would be better. I was referred to get an MRI scan by my GP [*general practitioner*] but when I came to the Hospital, they told me that we don’t have an MRI scan and you must go to the other hospital. When I went to that other hospital, I was put on a waiting list of more than six months, can you imagine, and I was in pain. I can die while I wait for an investigation to be done, honestly or government should make sure that they have got all these necessary equipment’s so that we don’t suffer.’ (P011)

**Subtheme 3.2: The lack of specialists in diagnosing varieties of chronic low back pain:** Chronic LBP is poorly diagnosed in KZN because of limited specialised personnel trained in diagnosing and treating musculoskeletal conditions such as chiropractic which has been shown to be effective in the treatment of CLBP. Because of limited accessibility of specialised personnel in primary care, CLBP patients are often not told what the problem is, how and why they have the problem. This misdiagnosis often results in inappropriate treatment culminating in chronic recurrent disabling back pain. One respondent said:

‘… So, the doctor gave me an injection and pills for LBP but when I was at home the pain kept getting worse. Oh, the time I was at the doctor he wrote a referral letter for me to come here …’ ‘When I got to the hospital, they did an X-ray and referred me to casualty and the first doctor to check me suspected it to be something to do with my bones. On the X-ray, the doctor told me that they couldn’t see anything, and I was discharged and given a prescription for medication Tramadol and Panadol, and I went home.’ (P012)

#### Theme 4: Treatment services

The PHC provides an optimum opportunity for the treatment of LBP as this approach incorporates health promotion, prevention and curative care. The PHC is a platform with a great potential to build long-lasting relationships between the community and health workers; thus, the PHC approach is best suited for the treatment of recurrent chronic disabling LBP. In spite of PHC’s suitability for the treatment of LBP, evidence has shown that LBP treatment in South African primary care settings is ineffective. Two subthemes emerge as main barriers to effective treatment of LBP.

**Subtheme 4.1: Communication barrier between healthcare professionals and patients:** Effective communication is key to the treatment of chronic pain condition such as CLBP. This study has shown that there is a lack of effective communication between the healthcare providers and patients. The healthcare system in KZN is overstretched by the burden of HIV and/or acquired immunodeficiency syndrome (AIDS) and tuberculosis and attention is given to HIV and/or AIDS patients who are the majority of the patients in primary care. On the other hand, because of work overload caused by disproportionate doctor–patient ratio or nurse–patient ratio necessitated by a high volume of HIV and/or AIDS patients, these healthcare providers find no time to educate patients. This results in a one-way model where the patients are not contributing to decide about their health. The use of medical terminology by other healthcare professionals also posed a potential barrier to effective CLBP treatment, because patients could not understand the language. One of the respondents mentioned that:

‘When I saw the doctor, they didn’t explain to me what exactly is going on, they just gave me sachet of painkillers and said some big words that I do not understand then she called the next patient to come into the consultation room. I was left amused and had to ask the nurse that was there if there is anything I needed to do to prevent the back pain from coming again. The nurse said to me if the doctor didn’t tell you anything else then it means there is nothing else you need to do besides taking these pills.’ (P013)

**Subtheme 4.2: Inadequate treatment of chronic low back pain condition:** The treatment of LBP in primary care is primarily by means of medication, and no other advice is given. This study found that the majority of CLBP patients are not satisfied with the care they receive from primary health. The main problem was the recurrence of symptoms, and some respondents reported that pain was not going away with medication. Therefore, ineffective treatment of acute episodes results in the progression to chronic recurrent pain, which can be disabling in some cases. Physiotherapy referral was rarely practised and this lack of a multidisciplinary leads to ineffective or inappropriate treatment or over-prescription of unnecessary medication. This approach contradicts with the guidelines for treatment of LBP and a departure from the multidisciplinary biopsychosocial model. Guidelines recommend multidisciplinary approach to the treatment of LBP, incorporating a wide variety of healthcare professionals, such as psychologists, neurologists, physiotherapists, chiropractors, exercise therapists and many more. The other respondents said:

‘I walked for about half an hour or 25 minutes it is not that long. So, when I arrived at the hospital, the doctor told me that it feels like pain related to my bones. He asked what type of work I do, and I mentioned that I usually sew clothes. The doctor explained that since I am always sitting down that is what has caused the LBP. He informed that I will be starting treatment because the back cannot be operated. The only treatment would be to take pills. I was then given a bag of painkillers; however, the pain did not go away. I ended up taking more pills than what I was told because I felt like they were not strong. Still the pain remained and that started to worry me as I was now thinking if I had cancer or any other serious problems going on.’ (P014)

‘Yes, I paid for transport the R300 and consultation with the private doctor. I told him that there is still no difference even after the injection and I went to the hospital, and they said that they did not see anything. He was surprised that they did not see anything because he was like its pain that the pills and injection are not solving so clearly there is something wrong. He injected me twice and gave me pills again but still no difference. He even wrote another referral letter to come back to Prince Mshiyeni and they transferred me to an out patient department (OPD). That’s where I found a doctor and I explained everything to him, and he read the doctor’s letter. He was surprised that how can the letter say LBP, but they are saying that they cannot find anything … he advised to go and get the old x-ray and do a new x-ray and bring them both. I did that, bear in mind at that point I am unable to walk because the time my back was affected my knees also collapsed as you can see …’ (P015)

## Discussion

The purpose of this study was to explore the patients’ lived experiences on the barriers to access diagnostic, referral and treatment services for CLBP. The approach to analysis was deductive, resulting in four preidentified themes and 13 subthemes that emerged namely: (1) the travel burden to the health facility, (2) long queues of waiting, (3) shortage of skilled personnel, (4) pyramidal pathway-induced delays, (5) unequal access to quality healthcare services because of a lack of universal insurance coverage, (6) delayed access because of sociocultural beliefs, (7) inadequate equipment, (8) the lack of specialists in diagnosing varieties of LBP, (9) poor technology resulting in an ineffective or inefficient process, (10) insufficient knowledge about the referral system, (11) social influence, (12) inappropriate/ineffective treatments, and (13) communication barrier leading to poor adherence.

To our knowledge, this is the first study in sub-Saharan Africa (SSA) to investigate the patients’ lived experience on the barriers to accessing diagnostic, referral and treatment services for CLBP. Studies done in high-income-countries (HICs) have focused on the barriers from the healthcare providers’ perspective.^27,28,29^ A study conducted in Australia investigated the patients’ and general practitioners’ perspective on the barriers to information provision in primary care.^29^ In a study conducted in the United Kingdom, McIntosh et al.^29^ showed that barriers to patient information provision exist and patients are dissatisfied with the information from their general practitioners (GPs) regarding diagnosis and treatment, thus patients tend to have other sources of information which contradict evidence contained in guidelines.

Nevertheless, the results of this study are congruent with evidence from HICs on similar subject. A scoping review on the factors that affect an effective referral system by Seyed-Nezhad et al.^30^ also identified technology as key to effective referral system. These authors further reported that electronic referral minimises unnecessary follow-ups for patients, improves confidentiality of patient information, increases access and reduces waiting times.^30^

The findings of Eide et al.^31^ concur with the findings of our study, which reported that unavailability of the required services or poor health infrastructure was one of the major barriers to accessing healthcare services. Although conducted in the context of cancer, a study in South Africa by Lubuzo et al.^32^ exploring patient access to healthcare services reported that distance to the healthcare facility was an important barrier to access. Their findings compare well with the results of our current study, which found that travel burden was a barrier to accessing PHC services for LBP patients. The travel burden encompassed cost, distance to the health facility and unavailability of quality and affordable services to the convenience of the patients. Our study found that beliefs around cause and effect from both the patient and the healthcare providers’ perspective affect timely access to care. This could be attributed to social norms, poor health-seeking behaviour or the acceptance that LBP is a normal life experience. We also found that shortage of trained healthcare professionals in PHC resulted in long waiting times to access care services. This is true for most underserved communities in LMICs and this shortage of community health workers (CHWs) could be attributed to several factors including, uncompetitive salary packages, poor infrastructure, poor living conditions and limited access to quality education.

Based on the findings of our study, the barriers to timely access of quality *diagnostic* services were based on use of poor-quality inadequate equipment, shortage of specialists and the brain drain in search for greener pastures.^33^ There is limited evidence on the patients’ perspective of the barriers to accessing diagnostic services for LBP. However, evidence on accessing diagnostic services of other health conditions such as TB^34,35^ and cancer exists.^32,35^ This study is in agreement with what was observed by Lubuzo et al.^32^ who reported that access to quality diagnostic services is limited because of inadequate knowledge and expertise of healthcare providers in primary care. Hartvigsen et al. reported that despite the advances in diagnostic technology, non-specific LBP is still a diagnostic enigma. Evidence has also shown that there is a poor correlation between spinal radiographic abnormalities and patients’ symptoms.^36,37^

Our study found that the treatment of CLBP is largely ineffective and does not conform to guidelines as it dwells on the traditional biomedical approach, which has shown limited effectiveness. Current guidelines recommend the use of a holistic multidisciplinary biopsychosocial approach to the treatment of CLBP.^38,39^ Over 90% of LBP patients in primary care are managed by pain medication and less than 15% go through to physiotherapy or rehabilitation.^38^ Our study could not find evidence that healthcare providers advise patients on self-care such as home stretches and exercises and preventive measures (such as avoidance of bad postural ergonomics including sitting posture, driving posture, office set ups, use of a back support and advice on sleeping position). This is consistent with the findings by Major-Helsloot et al.^38^ which reported that education, advice and exercise rehabilitation were not used enough, and treatments were ineffective. As the South African national development plan seeks to improve health outcomes by 2030, studies on healthcare providers’ non-adherence to guidelines behaviour are urgently needed to address this problem.^10^

The most important strength of this study was its ability to capture the patients’ lived experiences on the barriers associated with the care process for CLBP. This study relied on self-report data and it is always difficult to rule out recall bias. Additionally, this study was conducted in an urban setting and it is often reported that more challenges are found in rural setting, thus future studies should focus on barriers associated with CLBP care in rural settings of LMICs. Despite all these limitations, this study provides invaluable insights into the issues affecting LBP care in KwaZulu-Natal, South Africa. Furthermore, to the best of our knowledge, this is the first study in SSA that used qualitative methods to explore the patients’ lived-experiences regarding LBP care, an approach which is limited and needed in this field.

This study presents challenges faced by patients in receiving quality care for CLBP, a leading driver of disability. Based on the findings of this study, there is a need to avail resources to better understand CLBP and its management to inform policy. Thus, this study gives insight to healthcare decision and/or policy makers, funders and other involved stakeholders to ensure that there is sufficient allocation of healthcare resources. To improve LBP outcomes in South Africa, there needs to be a change of emphasis so that larger resources are allocated to LBP and other non-communicable diseases that pose a significant burden to the shoulders of the economy.

## Conclusion

This study shows that there are many barriers to accessing diagnostic, referral and treatment services for CLBP that need to be addressed in order to ensure quality healthcare delivery and an effective healthcare system. The shortage of skilled personnel and specialised skills in primary care posed a significant barrier to effective diagnosis and treatment of CLBP in KwaZulu-Natal and calls for improvements in the healthcare system including satisfactory remuneration of healthcare workers. This study also concluded that there is a need for universal health insurance coverage to ensure equity because a divide and an unequal access to quality healthcare services between the insured and the uninsured has been identified. Gaps in the referral system including the lack of adequate knowledge imply that there is insufficient health promotion. We have also concluded that the treatment of CLBP is ineffective, lacking the holistic multidisciplinary biopsychosocial approach.
